# Kinetics and Prediction of HBsAg Loss during Long-Term Therapy with Nucleos(t)ide Analogues of Different Potency in Patients with Chronic Hepatitis B

**DOI:** 10.1371/journal.pone.0098476

**Published:** 2014-06-06

**Authors:** Min-Ran Li, Hong-Li Xi, Qin-Huan Wang, Feng-Qin Hou, Na Huo, Xia-Xia Zhang, Fang Li, Xiao-Yuan Xu

**Affiliations:** Department of Infectious Diseases, Peking University First Hospital, Beijing, China; University of Cincinnati College of Medicine, United States of America

## Abstract

**Background & Aims:**

About 350–400 million people are infected with hepatitis B virus (HBV) chronically and 1 million people die of hepatitis B virus (HBV)-related liver diseases. Nucleos(t)ide analogues (NAs) have been used for the treatment against HBV. However, few studies have investigated the long-term effects of different nucleos(t)ide analogues on levels of hepatitis B surface antigen (HBsAg) in patients with chronic hepatitis B (CHB). The aims of this study were to measure the magnitude of HBsAg reduction by long-term monotherapy with adefovir dipivoxil (ADV) and entecavir (ETV), to compare HBsAg reduction between the two drugs of different potency and to predict the expected time needed to achieve HBsAg loss.

**Methods:**

We retrospectively evaluated the kinetics of HBsAg in 67 patients with CHB who all exhibited persistent viral suppression. These patients were treated with ADV or ETV for at least 6 years. HBV genotype was determined at baseline. Liver biochemistry, HBV serological markers, serum HBV DNA and HBsAg titers were determined at baseline, half year and yearly from year 1 to 6.

**Results:**

Serum HBsAg titers after treatment with ADV or ETV were significantly lower than the baseline titers (*P*<0.05). HBsAg reduction rate of patients treated with ETV (0.11 log_10_ IU/mL/ year) was higher than that treated with ADV (0.10 log_10_ IU/mL/year), and the calculated expected time to HBsAg loss for patients treated with ETV (approximate 24.99 years) was shorter than that with ADV (approximate 30.33 years), but there was no statistically significant difference between two groups (*P*>0.05).

**Conclusion:**

Serum HBsAg titers gradually decreased during long-term treatment with either ADV or ETV. It appears that the potency of ADV on HBsAg reduction is close to that of ETV, as long as patients have achieved persistent viral suppression.

## Introduction

Approximately one third of the global population have the evidence of past or present hepatitis B virus (HBV) infection and 350–400 million people worldwide are chronically infected with HBV. Moreover, 1 million die of the later stage of chronic hepatitis B (CHB)-related liver disease [1].

At present, nucleos(t)ide analogues (NAs) are the prevailing therapy for HBV infection. NAs include lamivudine (LAM), adefovir dipivoxil (ADV), telbivudine (LdT), entecavir (ETV) and tenofovir (TDF). Compared with LAM and ADV, ETV and TDF are more potent toward HBV DNA suppression [2]. The endpoints of therapeutic treatment include undetectable HBV DNA, hepatitis B e antigen (HBeAg) loss and seroconversion, hepatitis B surface antigen (HBsAg) loss and seroconversion. The ideal endpoint of antiviral therapy in CHB is HBsAg seroconversion. However, this is usually not achievable with the current therapy [3].

HBsAg is the surface antigen of HBV and serum HBsAg titers reflect the intrahepatic levels of the covalently closed circular DNA (cccDNA) and integrated HBV DNA sequence, which serve as the template for viral transcription [4]. Because of its long half-life, cccDNA is the major factor for HBV persistence and the limiting factor in the complete clearance of HBV infection. A number of recent studies have shown that ADV alone or plus pegylated interferon (PEG-IFN) can lead to a decreased level of intrahepatic cccDNA [5], [6], suggesting that these therapies may be useful to achieve the ideal endpoint of antiviral therapy for CHB.

The quantification of serum HBsAg titers, which has been recently advocated as another useful marker of disease activity, could add value to HBV DNA quantification and improve treatment monitoring [7]. HBsAg kinetics and prediction of HBsAg loss were demonstrated only during the short-term treatment with individual NAs [8], [9]. However, whether ETV or TDF is more potent toward HBsAg reduction than LAM or ADV remains unknown.

The aims of this study were to observe the kinetics of HBsAg in patients who had successful virological responses during long-term treatment with a less potent and a more potent NA, to investigate the potency of different NAs on HBsAg reduction, and to estimate treatment duration required to achieve HBsAg loss.

## Materials and Methods

### Patients

This retrospective study was performed at Department of Infectious Diseases of Peking University First Hospital (China). From September 2006 to December 2007, a total of 102 consecutive patients who were chronically infected with HBV were treated with either ADV or ETV. All patients were HBsAg-positive for at least 6 months prior to ADV or ETV treatment. Liver cirrhosis was diagnosed using clinical criteria [10]. All patients had not taken antiviral drugs (interferon alpha or NAs) for at least 6 months prior to ADV or ETV treatment. Inclusion criteria were as follows: monotherapy with NA (ADV or ETV) for at least six years, persistent viral suppression during continuous NA therapy. Exclusion criteria were as follows: partial virological response, presence of virological breakthrough because of poor compliance or drug resistance, switched to or added other drug, decompensated liver cirrhosis (Child-Pugh class B/C), hepatocellular carcinoma, liver transplantation, co-infections (hepatitis C virus or human immunodeficiency virus), liver disease that was not caused by HBV, and immunosuppressive treatment. Patients who had no available data at baseline and other testing time points were also excluded.

Patients received ADV at a dose of 10 mg daily. The treatment-naive patients received ETV at a dose of 0.5 mg daily. Other patients who had received LAM before study received ETV at a dose of 1.0 mg daily.

The study was in compliance with the Helsinki Declaration and was approved by the Medical Ethics Committee of Peking University First Hospital. All the enrolled patients gave their written informed consent.

### Laboratory assays

Liver biochemistry, HBV serological markers, serum HBV DNA and HBsAg titers were measured before treatment and 0.5, 1, 2, 3, 4, 5 and 6 years after NAs treatment.

Serum levels of alanine aminotransferase (ALT) were determined using an automatic biochemical analyzer [11]. HBV serological markers, including antibodies to HBsAg (anti-HBs), HBeAg and antibody to HBeAg (anti-HBe), were measured by ELISA kits (Abbott Laboratories, Chicago, IL, USA). Serum HBV DNA levels were determined using Cobas TaqMan assay (Roche Diagnostics, Basel, Switzerland), and the lowest limit of detection of the assay was 20 IU/mL. Serum HBsAg titers were measured using Elecsys HBsAg II quant assay (Roche Diagnostics, Branchburg, NJ, USA), with a linear range from 0.05 to 52000 IU/mL. HBV genotypes were determined by comparing the generated preS/S gene sequences (approximately 1,410 bp spanning nucleotide positions from 2825 to 1019) with prototype sequences from the Genbank using a web-based genotyping tool (NCBI)[12].

### Definitions of virological responses

According to European Association for the Study of the Liver (EASL) clinical practice guideline [13], virological response was defined as undetectable HBV DNA by a sensitive polymerase chain reaction (PCR) assay. Partial virological response was defined as the decrease in HBV DNA of more than 1 log_10_ IU/mL but detectable HBV DNA after at least 6 months of therapy in compliant patients. Viral breakthrough was defined as a confirmed increase in HBV DNA level of more than 1 log10 IU/ml compared to the nadir (lowest value) HBV DNA level on therapy.

### Statistical analysis

Statistical analyses were performed using the SPSS version 17.0 (SPSS, Chicago, IL, USA). We assumed that serum HBsAg titers followed a Log-normal distribution. Continuous variables were expressed as mean plus/minus standard deviation (SD) or median (interquartile range). Two-tailed Student's t-test or the Mann-Whitney U-test was used for statistical comparisons where appropriate. Categorical variables were compared by Chi-square or Fisher exact test. Repetitive measure analysis of variance (ANOVA) was used in analyzing HBsAg titers at different time points. The interaction between HBsAg reduction and drugs was assessed using Multi-variant test of repetitive measure ANOVA. The duration of treatment required to achieve HBsAg loss was estimated by applying a linear equation calculated by interpolating the median logarithmic decline over time for each single drug. The difference with *P* value of <0.05 was considered statistically significant.

## Results

From September 2006 to December 2007, a total of 102 patients with CHB were treated with ADV or ETV at our department. Forty one patients were treated with ADV, and 61 patients were treated with ETV. After extended treatment with NA, some patients were switched to other drug and some patients were treated with combination of drugs because of partial virological response or viral breakthrough. After applying the selection criteria described above, 20 (48.78%) patients with ADV and 47 (77.05%) patients with ETV maintained satisfactory virological suppression during at least 6 years of continuous monotherapy.

### Baseline characteristics

The baseline characteristics of the studied population are presented in Table 1. Among all patients, only 5 patients (7.46%) were with liver cirrhosis (Child-Pugh class A). The baseline variables, including age, the gender ratio, body mass index (BMI), HBeAg status, cirrhosis, ALT levels and HBV DNA levels, were not significantly different between patients treated with ADV and those treated with ETV (*P*>0.05). HBV genotype distributions among patients treated with ADV were B, C and undetermined in 3 (15.00%), 7 (35.00%) and 10 (50.00%), respectively, as compared with 5 (10.64%), 23 (48.94%) and 19 (40.42%) among patients treated with ETV (*P* = 0.565).

**Table 1:** Baseline characteristics of participated patients.

Characteristics	All patients (n = 67)	Adefovir dipivoxil (n = 20)	Entecavir (n = 47)	*P*
Age (years)	38.04±11.26	37.30±11.29	38.36±11.35	0.727
Male gender, n (%)	51(76.12%)	16(80.00%)	35(74.47%)	0.760
BMI	24.18±3.27	23.57±2.94	24.48±3.40	0.308
HBeAg-positive patients, n(%)	51(76.12%)	13(65.00%)	38(80.85%)	0.213
HBV genotype, n (%)				0.565
B	8(11.94%)	3(15.00%)	5(10.64%)	
C	30(44.78%)	7(35.00%)	23(48.94%)	
Undetermined	29(43.28%)	10(50.00%)	19(40.42%)	
CHB (%)	62(92.54%)	18(90.00%)	44(93.62%)	0.631
ALT>2×ULN, n (%)	49(73.13%)	16(80.00%)	33(70.21%)	0.408
HBV DNA(log_10_ IU/mL)	6.74±1.58	6.33±1.25	6.91±1.69	0.176
HBsAg (log_10_ IU/mL)	3.81±0.71	3.71±0.58	3.85±0.76	0.476

The variables were compared between patients treated with Adefovir dipivoxil and Entecavir.

### Changes in serum HBsAg titers

**Figure 1:**
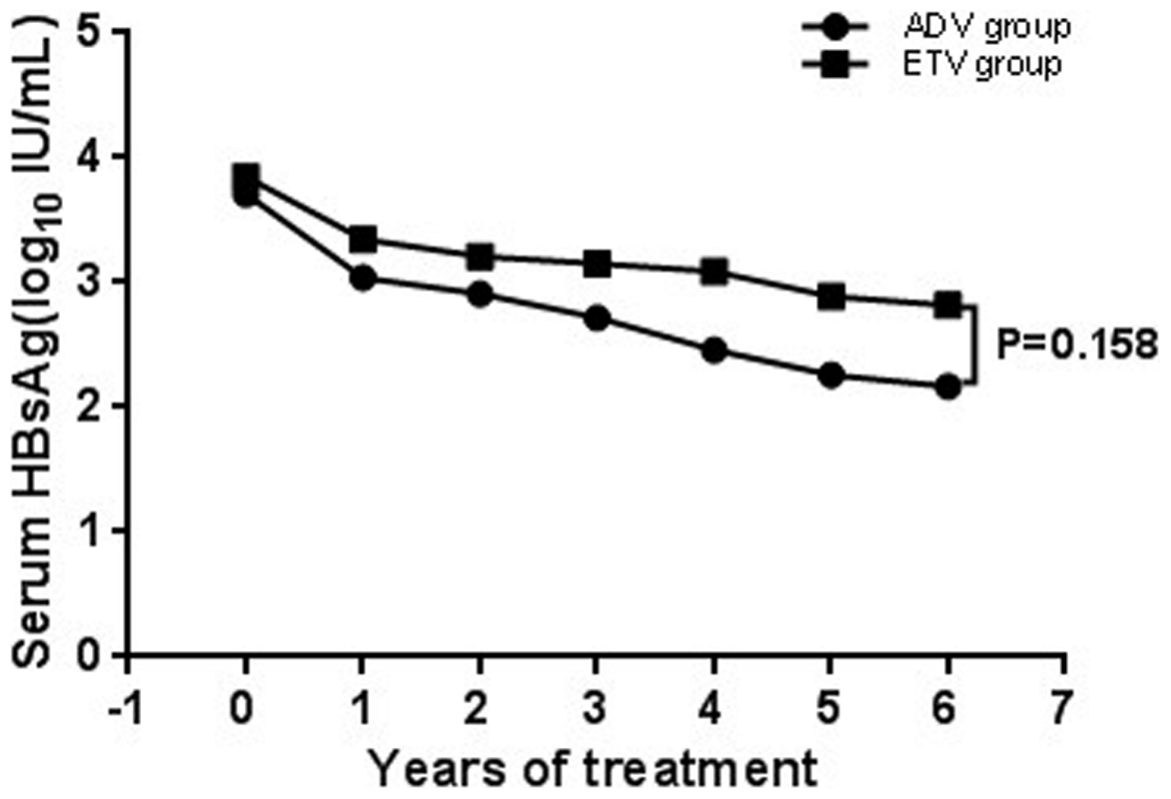
The mean HBsAg levels throughout a 6-year treatment period. In ADV and ETV groups, the mean HBsAg levels declined gradually, and HBsAg titers after treatment were significantly lower than the baseline titers (P<0.05). But there was no significant difference between the two drugs in HBsAg reduction levels during the 6-year treatment (P = 0.158).

The baseline HBsAg level in patients treated with ADV was not significantly different from that in patients with ETV (*P* = 0.476). The changes in serum HBsAg levels during treatment period are shown in Figure 1. Among the patients treated with ADV, HBsAg titers after treatment were significantly lower than the baseline titers (*P* ranged from 0.042 to 0.002). Among the patients treated with ETV, HBsAg titers after treatment were also significantly lower than the baseline titers (*P* ranged from 0.001 to 0.000007).

We observed that a median rate of HBsAg reduction during antiviral therapy was equal to 0.10 log_10_ IU/mL/year with ADV and 0.11 log_10_ IU/mL/year with ETV. No statistically significant difference in HBsAg reduction rate was observed between two drugs (*P* = 0.58). Multi-variant test of repetitive measure ANOVA analysis showed that two drugs were not significantly associated with HBsAg reduction levels (*P* = 0.158).

**Figure 2:**
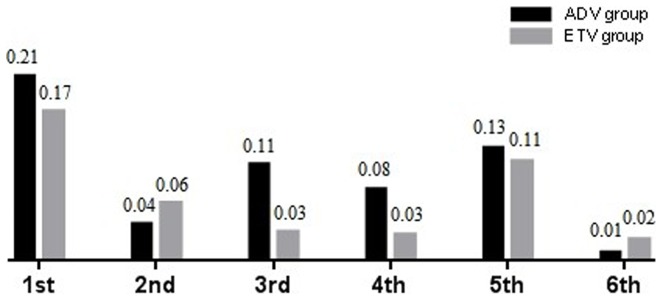
The median rates of HBsAg reduction in the 1st, 2nd, 3rd, 4th, 5th, and 6th year of treatment with ADV/ETV. Between the two drugs, no statistically significant difference was found in median rates of HBsAg reduction at the above time points.

The median rates of HBsAg reduction were 0.21, 0.04, 0.11, 0.08, 0.13, and 0.01 log_10_ IU/mL, respectively, after the 1st, 2nd, 3rd, 4th, 5th, and 6th year of treatment with ADV. The median rates of HBsAg reduction were 0.17, 0.06, 0.03, 0.03, 0.11 and 0.02 log_10_ IU/mL, respectively, after 1, 2, 3, 4, 5 and 6 years of treatment with ETV (Table 2, Figure 2). The rates of HBsAg reduction after the 1st, 2nd, 3rd, 4th, 5th, and 6th year of treatment showed no statistically significant difference between two drugs (*P*>0.05).

**Table 2:** The median rates of HBsAg reduction in the 1st, 2nd, 3rd, 4th, 5th, and 6th year of treatment with ADV/ETV.

	Adefovir dipivoxil	Entecavir	*P*
1st year	0.21	0.17	0.833
2nd year	0.04	0.06	0.762
3rd year	0.11	0.03	0.428
4th year	0.08	0.03	0.244
5th year	0.13	0.11	0.668
6th year	0.01	0.02	0.791

### Estimation of time to HBsAg loss during therapy

**Figure 3:**
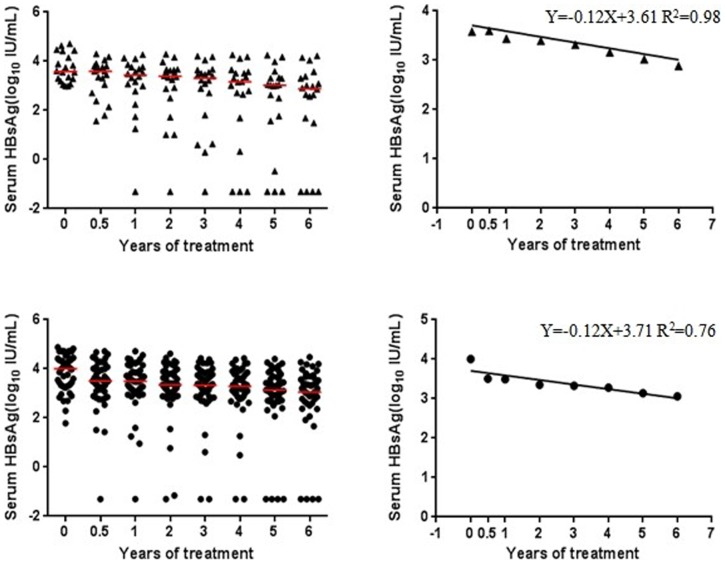
Prediction time (years) to HBsAg loss during ADV/ETV treatment. Scatter diagrams displaying the changes in HBsAg levels during therapy and the median levels of HBsAg at each time-point, linear equation calculated by interpolating the median logarithmic decline over time extrapolating the treatment duration required to achieve HBsAg loss during therapy. ▴-patients with ADV therapy; •-patients with ETV therapy.

HBsAg loss was observed in 4 of 41 (9.76%) patients treated with ADV and in 4 of 61 (6.56%) patients treated with ETV (*P* = 0.71). The linear regression formulations were Y = −0.12X + 3.61 (R^2^  = 0.98) for treatment with ADV, and Y = −0.12X+3.71 (R^2^ = 0.76) for treatment with ETV. The extrapolated expected time to HBsAg loss was nearly 30.33 (95% confidence interval (CI): 26.13–34.53) years for treatment with ADV, and 24.99 (95% CI: 12.53–37.44) years for treatment with ETV. The overlapping of 95% CI implied no statistically significant difference between the treatments with two drugs (Figure 3).

## Discussion

Both ADV and ETV have been approved for the treatment of patients with CHB for a number of years. Randomized clinical trials have shown that ETV is more potent than ADV in inhibiting HBV replication in CHB patients in early anti-viral therapy [14], [15]. Previous studies also examined the difference of the potency of NAs on HBsAg reduction. For instance, Boglione et al. [8] retrospectively evaluated different HBsAg kinetics in 134 treatment-naive patients treated with NAs therapy for 2 years. After 2 years of treatment, ADV therapy showed a decrease in HBsAg levels between LAM/LdT and ETV/TDF. The extrapolated expected time to HBsAg loss was nearly 31.1 years with ADV and 25.4 years with ETV. The patients in that study were only HBeAg-negative. Wong et al. [9] showed that the declined rate of HBsAg after 1 year of treatment was comparable between the more potent and the less potent NAs regardless HBeAg status. However, the observational duration of all the studies discussed here was only 1–2 years.

In this study, we retrospectively evaluated HBsAg kinetics in 67 Chinese patients with CHB who received at least 6 years of NAs therapy. This is the first study on kinetics of HBsAg in patients with a long-term suppression of HBV DNA by either ADV or ETV therapy. Since the patients had the same baseline characteristics, it allowed a comparative evaluation of HBsAg kinetics after ADV or ETV administration. While the study cohort included a few patients underwent previous treatment with interferon alpha or NAs, all of them had not taken any antiviral drugs for at least 6 months prior to ADV or ETV treatment. And patients who had received LAM before study received ETV at a dose of 1.0 mg daily. Thus, the previous treatment history and the dose of ETV (1.0 mg) may not significantly affect the results according to previous studies [16], [17]. Furthermore, the present study included only the patients who had responded favorably during NAs therapy. These included patients would be ideal to study the kinetics of HBsAg titers during the long-term NAs therapy and to predict the expected duration for HBsAg loss.

Our first important finding was that the decline in serum HBsAg titers in CHB patients during the long-term treatment with either ADV or ETV was significant. During ADV therapy, serum HBsAg titers were decreased at approximately 0.10 log_10_ IU/mL/year and nearly 30.33 years of treatment were estimated to be required for HBsAg loss. During ETV therapy, serum HBsAg titers were decreased at approximately 0.11 log_10_ IU/mL/year and nearly 24.99 years of treatment were estimated to be required for HBsAg loss. In our study, 8 (7.84%) patients achieved HBsAg loss, HBsAg titers were slowly decreased during NAs therapy in the remaining patients. Thus, HBsAg loss may occur after decades of NAs treatment. HBsAg is produced from the translated messenger RNAs of the transcriptionally active cccDNA and integrated HBV DNA sequence. NAs therapy not only inhibits HBV reverse transcription step, but also shows a small effect on the reduction of intrahepatic cccDNA [9], [18].

The second important finding was that although HBsAg reduction rate in patients with ETV therapy was higher than that with ADV therapy, and the calculated expected duration to HBsAg loss for patients treated with ETV was shorter than that for patients treated with ADV, there was no statistically significant difference between two groups, implying that the potency of ADV on HBsAg reduction is similar to that of ETV. These results are not surprising because they are similar to those reported by Boglione et al. [8], who showed that ADV had a better serological response than virological response. The potency of LAM on HBsAg reduction was also demonstrated by the study of Seto et al. [19], who showed a similar median HBsAg decline rate (0.10 log_10_ IU/mL/year) during a 10-year treatment with LAM in patients who had responded to LAM favorably. Both previous studies and our study included patients with the same character, i.e. they all had responded favorably to NAs. Therefore, it appears that HBsAg decline rate of patients treated with less potent NAs (LAM, ADV) is similar to that of patients treated with the more potent NAs (ETV), as long as they have achieved continuous virological suppression. One likely explanation of this finding is that the reduction rates of cccDNA levels after long-term antiviral therapy with the more potent and the less potent NAs are similar, because previous studies showed that cccDNA decline rates were also comparable between two types of NAs drugs [18], [20]. This inference does not include TDF, because the previous study by Boglione et al. [8] showed that TDF reduced HBsAg levels more significantly than ADV. However, it is still unclear why only TDF possess the superior reduction capability over ADV, whereas ETV does not. One possible reason is that TDF has a stronger antiviral effect against HBV than ETV does [21].

Some limitations of this study need to be considered. Several studies have explored the factors that are significantly associated with HBsAg reduction, such as HBeAg status, HBV genotype, and HBV DNA levels [22]–[24]. Because of the relatively small number of patients, our study was limited to assess the variables that are significantly associated with HBsAg reduction during ADV/ETV therapy. And because more patients with ADV therapy had partial virological response or viral breakthrough than ETV, the numbers of patients between two groups were not well balanced. This may have influence on the comparison between two drugs. The large-scale studies with more patients are needed to validate these findings in the future. In addition to ADV/ETV, LAM/LdT has also been used extensively for patients with CHB. However, because of high mutation rate or side effect, a few patients who were treated with LAM/LdT monotherapy for at least six years and had satisfactory virological suppression were recruited. Thus, those patients were not included in this study.

In summary, serum HBsAg titers were continuously decreased during the long-term treatment with either ADV or ETV, but HBsAg decline rate and the extrapolated expected time to HBsAg loss were not significantly different between ADV and ETV therapies. Thus, it appears that the potency of ADV on HBsAg reduction is close to that of ETV among patients who have achieved persistent viral suppression during the long-term antiviral treatment.
